# Explicit atomistic modelling of solid–solid interfaces: from construction to design

**DOI:** 10.1039/d6cc02091g

**Published:** 2026-07-21

**Authors:** Anastasia K. Lavrinenko, James A. Quirk, James A. Dawson, Marnix Wagemaker, Alexandros Vasileiadis

**Affiliations:** a Storage of Electrochemical Energy, Department of Radiation Science and Technology, Faculty of Applied Sciences, Delft University of Technology Mekelweg 15 Delft The Netherlands a.vasileiadis@tudelft.nl; b School of Engineering, Physics and Mathematics, Northumbria University Newcastle upon Tyne NE1 8QH UK; c Chemistry – School of Natural and Environmental Sciences, Newcastle University Newcastle upon Tyne NE1 7RU UK

## Abstract

Solid–solid interfaces play a critical role in the performance of energy storage systems, catalysts, and electronic devices, yet their atomic-scale structure and behaviour remain challenging to probe experimentally. Explicit atomistic modelling provides a powerful approach for investigating interfaces by directly constructing and simulating contacting phases, enabling the calculation of interfacial energetics, electronic structure, charge transfer, stability, and transport processes. This Review summarises the methodology and applications of explicit interface modelling, including interface construction strategies, properties accessible from atomistic simulations, and recent advances enabled by machine-learned interatomic potentials that extend simulations to larger time and length scales. We also discuss how computational results can be interpreted and validated using experimental characterisation techniques, and highlight current challenges and emerging opportunities in predictive interface modelling and materials design.

## Abstract 2

How an atomistic interface model is built decides its reliability. This Review follows the workflow from model construction and property extraction with DFT, AIMD and machine-learned potentials to experimental characterization and interface design.

## Graphical abstract

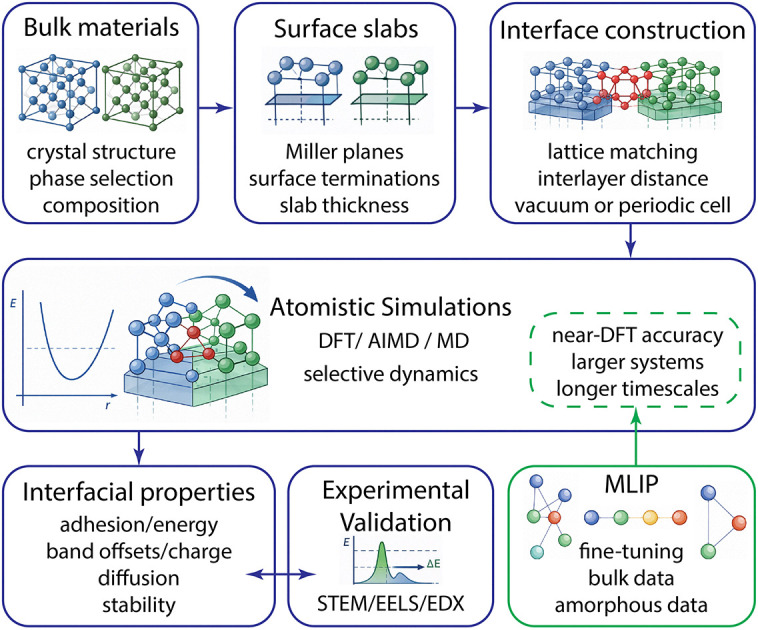

## 1. Introduction

Atomic-scale structure and electronic effects at the interfaces govern the performance of macroscopic devices and determine their properties. In all-solid-state batteries, interfaces between cathode active materials and solid electrolytes determine impedance, cycling stability, and safety.^1,2^ In heterogeneous catalysis, substrate/catalyst contacts create unique active sites where charge transfer tunes selectivity.^3^ In semiconductor devices, band offsets at heterojunctions control carrier injection and transport.^4^ Despite their importance, interfaces remain extremely difficult to characterize experimentally. Interfaces are localised structures often only a few nanometres in width, which limits techniques to those able to spatially resolve fine details, such as electron microscopy, which requires good-quality samples prepared by cutting lamellae from thin films with a focussed ion beam. Even if we have access to an electron microscope and a good quality sample – and are fortunate enough to have an interface visible in our lamella – we still must contend with these high energy analysis and preparation techniques damaging samples and confounding results.^5^ Computational methods, including density functional theory (DFT), *ab initio* molecular dynamics (AIMD), and classical molecular dynamics (CMD), enable us to explore interfacial phenomena at the atomic level and provide important insights into experimental characterization and interfacial understanding, without the physical constraints of sample preparation and measurement.

**Fig. 1:**
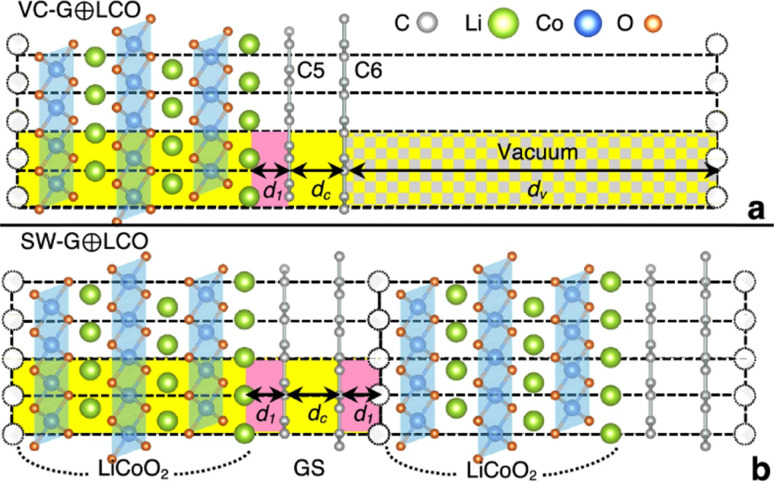
The schematic structures of graphene stack (GS) (001) and LiCoO_2_ interface: (a) vacuum model; (b) periodic sandwich model. Interlayer distance between carbon and LiCoO_2_, two carbon layers are shown as *d*_1_, *d*_c_, respectively, while *d*_v_ indicates the vacuum distance. The primitive cell is indicated by the black dashed lines, and calculated supercells are shown in the yellow highlight area. The grey, green, blue, orange, and white balls denote carbon, lithium, cobalt, oxygen, and missing lithium atoms, respectively. Reprinted from ref. 6 Copyright 2024 Springer Nature, licensed under CC BY 4.0.

Unlike bulk materials, interfaces are structurally and chemically heterogeneous, often containing local strain, defects, off-stoichiometry, and non-equilibrium bonding environments. These features make their behaviour difficult to infer from bulk descriptors alone and motivate the use of explicit interface models in which both phases are represented atomistically in direct contact (Fig. 1).

Explicit interfacial modelling allows local bonding environments, band alignments, interfacial energetics, charge redistribution, and diffusion processes to be evaluated directly, offering a practical route to connect atomic-scale mechanisms with experimentally measurable behaviour. However, the field remains relatively young, with no universally adopted methodology and a need to establish best practices for interface construction and modelling.^7,8^

**Fig. 2:**
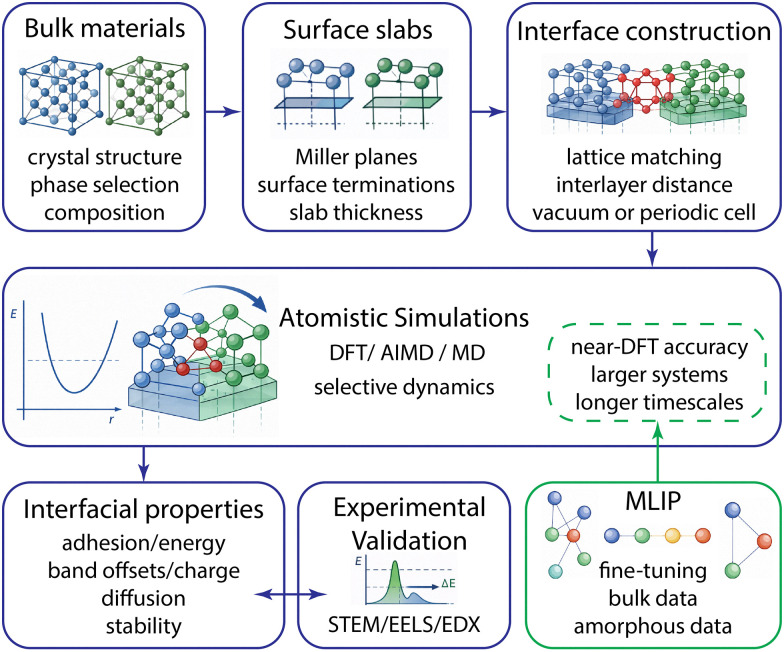
Workflow of explicit atomistic modelling of solid–solid interfaces, from bulk materials and surface slab construction to interface modelling, atomistic simulations, extraction of interfacial properties, machine-learned interatomic potentials, materials screening and design, and experimental validation. Each step involves key modelling decisions and considerations that determine the reliability and predictive power of the interface model.

Explicit atomistic interface modelling can capture properties that are inaccessible from bulk or surface calculations, yet their accuracy depends on choices made during the structural model construction. This Review outlines a practical modelling workflow and highlights the model-building choices that strongly affect the reliability of calculated properties. We show that properties accessible from static DFT calculations can already provide useful insights into interface energies, band alignment, charge transfer, and thermodynamic stability. However, slower temperature-dependent processes such as ion transport across the interface or structural rearrangements require simulations over time with timescales that remain inaccessible to AIMD. We discuss how machine-learned interatomic potentials (MLIPs) can extend simulations to the time and length scales needed to study disorder, interfacial reactions, and kinetic processes at interfaces, although the quality of the underlying first-principles training data remains a limiting factor. We also relate these calculated properties to experimental characterization techniques such as scanning transmission electron microscopy, electron energy loss spectroscopy, and electrochemical impedance spectroscopy. Finally, we summarize the main limitations of current interface models and discuss how explicit atomistic interface modelling can support interface design. An overview of this workflow is illustrated in Fig. 2.

## 2. Constructing explicit interface models

Constructing a reliable interface requires setting multiple parameters that affect the computed properties. The first is the choice of crystallographic planes and the surface termination which affect both the thermodynamics and kinetics of interfacial reactions. For example, on MgV_2_O_4_ spinel cathodes, the V–O terminated (001) surface has lower surface energy and provides more accessible adsorption sites for solvated Mg electrolytes than the V-terminated (111) surface, leading to activation barriers for desolvation and intercalation that differ by several eV and estimated diffusivities that vary by tens of orders of magnitude between the two orientations.^9^ The Materials Project Crystalium database^10^ provides pre-computed Wulff shapes for over 100 polymorphs. Low-index surfaces are preferred because they minimise the surface energy and dominate the equilibrium crystal shape. However, in practice, interfaces may form along higher-index planes dictated by processing conditions, grain orientations, or epitaxial relationships, making the plane selection itself a modelling choice that should be justified by experimental parameters or surface energy arguments.

Two crystals rarely share identical lattice constants, and finding a common supercell that minimises strain is essential. The Zur and McGill algorithm^11^ is widely used. It generates all transformation matrices up to a maximum supercell area and filters by length and angle mismatch. The surface mismatch parameter is calculated as:
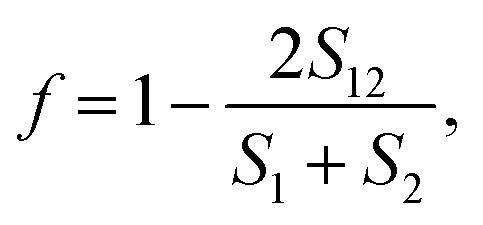
where *S*_1_ and *S*_2_ are the surface areas of the slabs and *S*_12_ is the overlapping area between their surfaces.^12^

Each material slab must be thick enough to include a bulk-like electronic structure. The slab thickness is converged by monitoring the surface energy (or energy of the slab *E*_slab_) with an increasing number of layers.^13,14^ Surface energy qualifies the stability of the surface relative to bulk materials and can be calculated as follows:
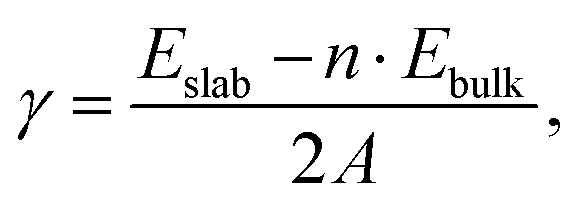
where *E*_slab_ is the total slab energy, *n* is the number of bulk formula units in the surface slab, *E*_bulk_ is the energy per formula unit from a slab-consistent bulk cell, and *A* is the surface area of one surface. The factor of 2 accounts for both exposed surfaces of a symmetric slab. For asymmetric slabs where the two terminations differ, this expression yields an average of the two surface energies rather than the energy of either individual interface.^15–19^

The equilibrium interlayer distance between the two materials is determined by shifting the film along the *z* direction over a range of 1.5–6.0 Å and recording the total energy at each interlayer distance. The resulting binding-energy curve exhibits a minimum at the equilibrium distance.^20,21^ Binding energy is defined as:
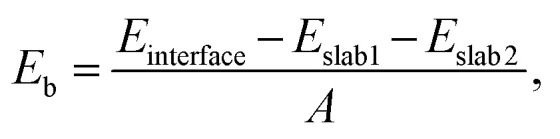
where *E*_interface_ is the total energy of the interface, *E*_slab1_ and *E*_slab2_ are the energies of the isolated slabs computed with the same in-plane strain as in the interface model, thereby excluding the mechanical contribution of lattice matching, and *A* is the interfacial area. In fully periodic models without a vacuum, the supercell contains two interfaces. In this case *A* then represents their combined area, and *E*_b_ yields the average binding energy of the two junctions.

Whether to include a vacuum layer depends on the target property. The slab-vacuum model inserts 15–20 Å of vacuum along the *z* direction, preventing interaction between periodic images (Fig. 1a). It is suited to surface properties, adsorption, and thin-film studies.^15^ The fully periodic sandwich model eliminates vacuum (Fig. 1b) and is preferred for bulk interface energetics, grain boundaries, and band-offset calculations. It requires symmetric stacking to cancel net dipoles.^22,23^ In both models, 2–4 bottom layers of the material may be frozen *via* selective dynamics to their bulk positions to mimic a semi-infinite material, while at least 2–3 layers on each side of the interface are relaxed freely.^24^ Without selective dynamics, thin slabs undergo unphysical relaxation.

In practice, pymatgen^25^ and ASE^26^ provide comprehensive tools for interface construction, from lattice matching and slab generation to selective dynamics assignment, while VASPKIT^27^ offers efficient post-processing of interface AIMD trajectories, including extraction of charge density difference, band structures, and layer-resolved electronic structure.

## 3. Properties accessible from explicit interface models

The power of explicit interface modelling lies in the properties it delivers.

### 3.1 Work of adhesion

Work of adhesion quantifies interfacial bond strength. Positive *W*_ad_ indicates a thermodynamically stable interface that requires energy to separate the two contacting surfaces to an infinite distance. Work of adhesion is defined as:
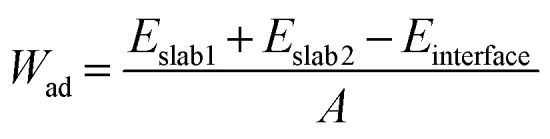
where *E*_slab1_ and *E*_slab2_ are the total energies of the two isolated slabs strained to the interface lattice parameter, thereby excluding the mechanical contribution of lattice matching, *E*_interface_ is the total energy of the interface supercell, and *A* is the interfacial area.^12,17,28,29^ In fully periodic models without a vacuum, *A* represents the combined area of two interfaces, and *W*_ad_ yields the average work of adhesion of the two junctions. In a systematic DFT study of about 20 solid–solid interfaces, *W*_ad_ was shown to be an important descriptor for evaluating interfacial stability, revealing that it largely depends on the surface orientation and termination.^30^ For anode|electrolyte interface models, DFT-calculated interfacial formation energies, corresponding to the negative work of adhesion, were compared with experimental electrochemical activity and cycling stability across a series of Mg alloy anodes.^31^ The authors showed that interfacial formation energy should be considered together with the phase topology and electrochemical kinetics of the secondary phase to identify favorable alloy compositions.

### 3.2 Interface energy

The interface energy (*γ*_int_) follows from the Dupre relation describing the energy per unit area of creating two free surfaces from the energy recovered upon bonding:*γ*_int_ = *γ*_slab1_ + *γ*_slab2_ − *W*_ad_where *γ*_slab1_ and *γ*_slab2_ are the surface energies of the slabs.^28,32^ When the two lattices are mismatched, this expression includes an artificial strain contribution that increases with slab thickness. In several studies, this contribution was corrected either by fitting a series of supercells with increasing slab thickness and extracting strain-free interface energy from the intercept of a linear fit^17^ or by explicitly subtracting the strain energy.^20,33^ After this correction, *γ*_int_ reflects the chemical interaction at the interface rather than the strain imposed by the periodic supercell.

A negative *γ*_int_ indicates that forming the interface is energetically favorable with respect to the chosen bulk references. Such values are often associated with chemically active interfaces, where strong interactions across the contact can lead to structural rearrangement, interdiffusion, or decomposition.^17,34^ For example, negative *γ*_int_ values reported for sulfide solid electrolyte interfaces with Li metal were attributed to chemical instability of the interfaces consistent with the anion decomposition observed in AIMD trajectories.^20^

Positive *γ*_int_ values indicate that interface formation has an energy cost with respect to the chosen bulk references. Among positive values, smaller *γ*_int_ generally corresponds to more favorable contact and better wetting, whereas larger *γ*_int_ indicates less favorable and chemically inert contact between the two surfaces.^17,20,34^ In the Li/solid electrolyte interfaces, LiF had the highest positive interfacial energy which the authors associated with poor wettability toward Li metal but stronger resistance to dendrite penetration through the solid electrolyte interphase (SEI).^35^*γ*_int_ should therefore be interpreted as a thermodynamic stability indicator since kinetic barriers can still stabilize interfaces that interface energy analysis would suggest to be reactive.^17^

### 3.3 Band offsets

Band offsets at semiconductor and battery interfaces can be extracted using the model-solid theory from three DFT calculations, which are two bulk and one interface:Δ*E*_v_ = (*E*^2^_v_ − *E*^1^_v_) + Δ*V*,where *E*^1^_v_ and *E*^2^_v_ are valence band maxima with respect to the average electrostatic potential in materials 1 and 2, respectively, and Δ*V* is the difference in average potential between the two bulk-like regions extracted from the interface calculation.^14,36^ This property captures the actual charge transfer and dipole formation at the junction.^13^ The conduction band offset can be determined using band-gap values *E*^1^_g_ and *E*^2^_g_:Δ*E*_c_ = Δ*E*_v_ + *E*^2^_g_ − *E*^1^_g_

This method applied to GaN/AlN interfaces revealed that large polarisation-induced electric fields created a strain-dependent asymmetry of band offsets.^37^ More recently, the process was automated. The InterMat was developed by combining the Zur matching algorithm with a graph neural network force field to generate a dataset of 183921 interface band offsets and 607 surface work functions, accelerating semiconductor interface screening.^38^

### 3.4 Projected density of states

The projected density of states (PDOS) resolved onto atomic layers provides a complementary view of interfacial electronic structure. By decomposing the DOS, such parameters as the spatial extent of band bending, the formation of interface states within the gap, electronic transport, and the local narrowing or broadening of bands due to hybridisation can be identified.^12,14,17,18,21,29,39^ For the LiCoO_2_/β-Li_3_PS_4_ interface, layer-resolved PDOS was used to show that the Li chemical potential step across the junction drives Li accumulation on the electrolyte side, forming a resistive space-charge layer.^40^ Experimentally, electron energy loss spectroscopy (EELS) is a very useful technique, as it can identify the oxidation state of elements and even make band gap measurements at an interface,^41^ if a suitable thin film of the sample can be produced. Such experiments have poor spatial resolution, however, and can be complex to analyse.

### 3.5 Charge redistribution

Charge redistribution at interfaces is often analysed using Bader charges. Since atomic charges are not direct quantum-mechanical observables, the electron density obtained from DFT must be divided among atoms using a defined partitioning scheme.^42^ In Bader analysis, space is partitioned into atomic volumes separated by zero-flux surfaces in the charge-density gradient, and the charge assigned to each atom is obtained by integrating the electron density within its volume.^43^ This makes Bader analysis insensitive to the basis set and well suited to plane-wave DFT calculations. In contrast, the commonly used Mulliken analysis is sensitive to basis-set choice and is not applicable to plane-wave basis sets that carry no atomic functions.^44^ The grid-based algorithm developed by Henkelman and co-workers made this approach practical for large supercells by assigning charge-density grid points to Bader volumes through steepest-ascent paths on the grid.^42^

**Fig. 3:**
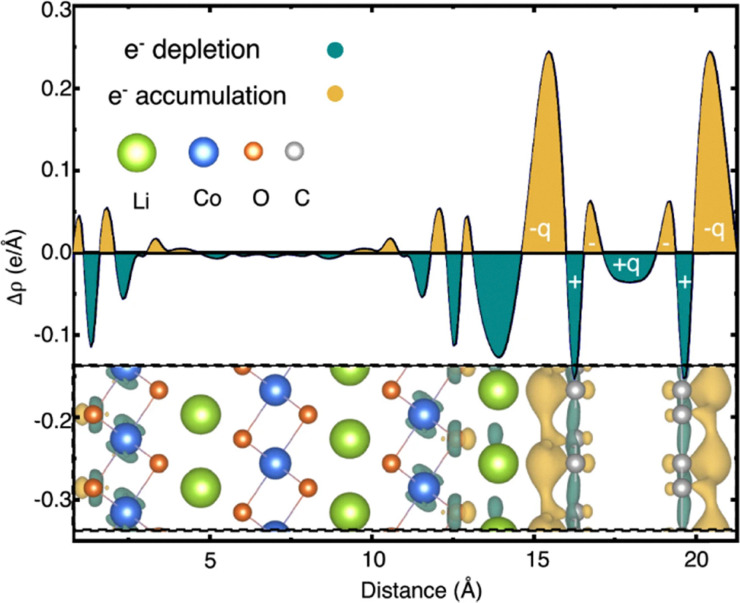
Calculated plane-averaged charge density difference. The corresponding isosurfaces of charge density difference is the inset. Orange (green) region represents charge accumulation (depletion). Reprinted from ref. 6 Copyright 2024 Springer Nature, licensed under CC BY 4.0.

Charge redistribution at the interface revealing electron accumulation or depletion is obtained as^18,39,45,46^Δ*Q* = *Q*_interface_ − *Q*_slab1_ − *Q*_slab2_,where *Q*_interface_, *Q*_slab1_, and *Q*_slab2_ are the Bader charges summed over all atoms in the interface supercell, isolated slab 1, and isolated slab 2, respectively. Positive Δ*Q* indicates electron accumulation, while negative indicates depletion. Visualised as charge density difference isosurfaces (Fig. 3), this reveals interfacial dipole formation and electron accumulation zones. For MgV_2_O_4_ slab models, Bader analysis was used to examine charge redistribution at the cathode surface after water adsorption.^9^ At one monolayer coverage, it was found that approximately 0.4*e* is transferred from the MgV_2_O_4_ surface to the adsorbed water layer. The resulting interfacial electrostatic field was linked to a lower activation energy for Mg electrolyte desolvation and intercalation.

Experimentally, X-ray photoelectron spectroscopy (XPS) depth profiling can track changes in oxidation states across the interface region providing evidence for interfacial charge transfer and phase reconstruction during electrochemical cycling.^31^ The depth resolution of the technique is limited, however, by preferential sputtering and ion-beam-induced mixing,^47^ making quantitative comparison with computed charge density difference maps at the interface difficult.

### 3.6 Electrochemical stability

Electrochemical stability and phase decomposition at interfaces are mostly assessed through a thermodynamic approach based on bulk materials calculations, in which reaction energies between contacting phases are evaluated from their respective convex hulls.^48^ Within this approach, an interface is thermodynamically stable only if no product phase is energetically more favorable than the contacting materials at the given electrochemical potential. In practice, only a few interfaces satisfy this condition. Many interfaces that operate successfully in devices are instead kinetically stabilized, meaning that decomposition is thermodynamically favored but proceeds slowly because the associated reaction barriers are high.

Although thermodynamic calculations are valuable for identifying possible decomposition products and reaction driving forces, they do not determine whether the resulting interface will be passivating or continue to decompose. This limitation is well known for bulk solid electrolytes, for which computed electrochemical stability windows are far narrower than those measured experimentally.^49–51^ The apparent wider stability arises from kinetic passivation by decomposition products rather than from intrinsic thermodynamic stability. In the case of kinetic passivation, the decomposition products form a thin interlayer that is electronically insulating but ionically conducting thereby suppressing further reaction.

Predicting whether a given interphase passivates or propagates, therefore, requires modelling of kinetic interface evolution, rather than relying on static thermodynamic assessment alone.^7,17,52^ This is becoming increasingly feasible through MLIPs, which extend near-DFT accuracy to the system sizes and timescales needed to observe passivation dynamics directly.^53,54^

### 3.7 Interfacial stability

**Fig. 4:**
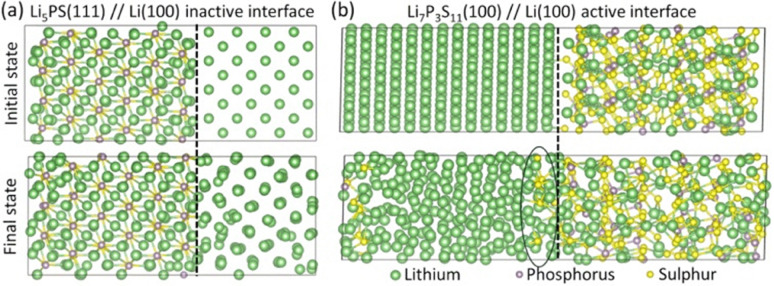
The initial (upper) and the final (lower) states of (a) chemically inactive Li_5_PS//Li, and (b) chemically active Li_7_P_3_S_11_//Li interface structures. The dashed black line indicates the initial position of the interface. The black oval highlights the sulphur diffusion into lithium metal. Reprinted from ref. 19. Copyright 2025 Royal Society of Chemistry, licensed under CC BY 3.0.

Interfacial stability and reconstruction can be assessed directly through AIMD by monitoring whether chemical reactions occur during the simulation.^18–20,29,55,56^ Structural evolution is tracked by comparing radial distribution functions and coordination numbers with those of the reference bulk phases, enabling detection of bond breaking, changes in coordination environment, and new phase formation. Although AIMD timescales are very short compared to practical operation, they are sufficient to distinguish relatively stable from reactive interfaces and to identify the initial decomposition products that govern interphase growth. For example, a Li_5_PS|Li interface remained stable during 100 ps of AIMD, whereas electrolytes Li_7_P_3_S_11_ and Li_3_PS_4_ under the same conditions showed rapid SEI layer growth, driven by the dissolution of PS_4_^3−^ and P_2_S_7_^4−^ units (Fig. 4).^19^ Notably, however, AIMD can demonstrate interfacial degradation, but it cannot conclusively prove that an interface is stable. The absence of reactivity within a simulation may simply reflect insufficient simulation time rather than interfacial stability. Longer timescales accessible through MLIPs are ultimately needed to distinguish passivated interfaces from those whose decomposition has not yet been observed.

Experimentally, either EELS or energy dispersive X-ray spectroscopy (EDX), are able to produce spatially resolved chemical maps that can identify elemental interdiffusion in the interface region,^57^ providing an excellent means to confirm computational predictions of interface reactions, but both methods can struggle with light elements such as hydrogen or lithium. Another alternative is secondary mass ion spectrometry (SIMS), in which a surface is sputtered, then the ejected secondary ions are measured. SIMS has the advantage of extreme sensitivity to light ions that may elude detection with EELS, as well as the ability to produce 3D composition profiles, but it is an inherently destructive technique.

### 3.8 Ion transport across interfaces

Ion transport across interfaces is quantified by the diffusion coefficient calculated *via* the Einstein relation:^19,30,58^
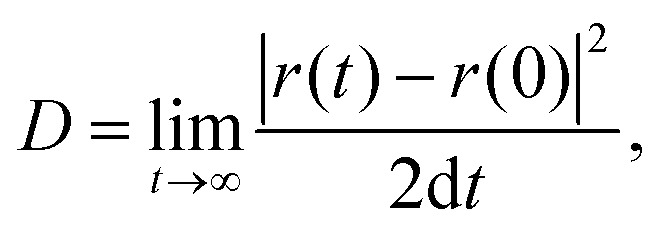
where 〈|*r*(*t*) − *r*(0)|^2^〉 is the mean squared displacement (MSD) of the diffusion ions at time *t*, and *d* is the diffusion dimensionality. The diffusion coefficient yields activation energies through Arrhenius fitting of *D*(*T*) across multiple temperatures^30^ and enables direct comparison of ion transport between bulk-like and interfacial regions, as well as resolution of anisotropic transport along individual crystallographic directions.^14,18,21^ Besides ion transport evaluation, the MSD of individual species crossing the interface has been used to classify interfaces as stable, passivating, or reactive.^52^

Where spatially resolved barriers are needed, the nudged elastic band (NEB) DFT method maps the minimum-energy pathway for ion migration across specific interfacial sites, providing activation energies for individual hopping events and, when applied to decomposition, for bond-breaking reactions at the interface.^17,39^ NEB calculations have been applied to determine the kinetic activation barriers for Mg^2+^ site-hopping from the cathode surface into the bulk after desolvation of the electrolyte.^9^ Surface conditions such as magnesiation state, crystallographic orientation, and transition-metal doping of MgV_2_O_4_ slab were found to modify the barrier of rate-limiting steps along the desolvation–intercalation pathway by up to several eV. Similarly, NEB calculations were used to compute Mg migration barriers for Mg adsorption configurations on bare Mg(101) and Mg_3_Bi_2_(011) anode surfaces.^59^ The calculations showed stronger Mg adsorption and a lower diffusion barrier on Mg_3_Bi_2_ than on bare Mg. These results indicate that Mg_3_Bi_2_ is more magnesiophilic and facilitates surface Mg diffusion, explaining the experimentally observed preferential deposition on Mg_3_Bi_2_ eutectic regions and the resulting bubble-like SEI morphology.

The ability to spatially resolve activation energies and attribute them to specific processes is a key strength of computational methods. Ionic diffusion through an interface has been measured by electrochemical strain microscopy where interfaces terminate at a surface, but the spatial resolution is poor, and it precludes study of internal grain boundaries.^60^ More often, electrochemical impedance spectroscopy (EIS) is employed, which aims to decompose total resistance into contributions from bulk, grain boundaries, heterogeneous interfaces, *etc.* This requires fitting data to a hypothetical circuit, which is non-trivial when spectral features overlap, as distinct circuits can reproduce the same spectra.^61^ Even if the hypothetical circuit is reasonable, it will not explain the origin of the resistance.

## 4. Machine-learned interatomic potentials for interfaces

Although DFT and AIMD provide valuable insights into interfacial phenomena and can match computed properties with experimental results, they are highly computationally expensive for interface modeling and are limited to hundreds of atoms and tens to hundreds of picoseconds. These times and scales are not enough to fully assess interface evolution and stability, particularly for amorphous interphases, polycrystalline grain boundaries, and slow kinetic processes such as cation intermixing. MLIPs are now pushing this cost–accuracy barrier further. The latest equivariant graph neural network architectures, such as MACE,^62^ NequIP,^63^ and Allegro^64^ achieve state-of-the-art accuracy while scaling to millions of atoms. Universal MLIPs trained on broad databases (M3GNet,^65^ CHGNet^66^) enable rapid screening of novel interface chemistries without system-specific training. However, a recent benchmark revealed that these universal models exhibit significant errors for surface properties, because their training data are mostly bulk structures.^67^ Fine-tuning of universal models on surface- or interface-specific DFT data, whether through transfer learning or active learning, substantially recovers accuracy lost in the original training. Active learning, where model uncertainty triggers targeted DFT calculations, is critical for efficiently sampling the vast configurational space of interfaces for further MLIPs development.^68,69^

Recent studies have highlighted the growing role of MLIPs in interface modelling. A comprehensive review summarizes the application of MLIPs to model surfaces, solid–solid interfaces, and solid–liquid interfaces.^70^ Another review focuses on MLIP-based modelling of interfaces in all-solid-state batteries providing practical guidance on training-data strategies, model selection, and validation.^69^ It emphasizes that training datasets benefit from the inclusion of amorphous and disordered configurations and may not require explicit interfacial structures to represent them accurately. It also highlights that MLIP validation should extend beyond reproducing forces and energies to include physically meaningful properties such as radial distribution functions, diffusion coefficients, voltage profiles, phase stability, or interfacial energies extracted from reference DFT and AIMD calculations or experimental measurements. This strategy was demonstrated for the Li–Si anode system. A DeePMD model was trained on crystalline Li, Si, and Li_*x*_Si phases together with amorphous Li_*x*_Si structures across the full compositional range, without any interface structures in the training set.^71,72^ The model reproduced the experimental voltage plateau difference between crystalline and amorphous Si lithiation and the voltage hysteresis associated with the crystalline-to-amorphous Li_3.75_Si phase transformation.^71^ The model was then applied to Si/Li interface structures that were not seen during training. It yielded low energy and force errors and correctly captured the experimentally observed anisotropy of lithiation lengths across different Si facet orientations.^72^ The application of MLIPs has also been extended to amorphous materials and their interfaces^53,73^ demonstrating that MLIPs can be used to study larger and more structurally complex interface models than are accessible with direct first-principles methods.

## 5. Where experiment and theory support each other

**Fig. 5:**
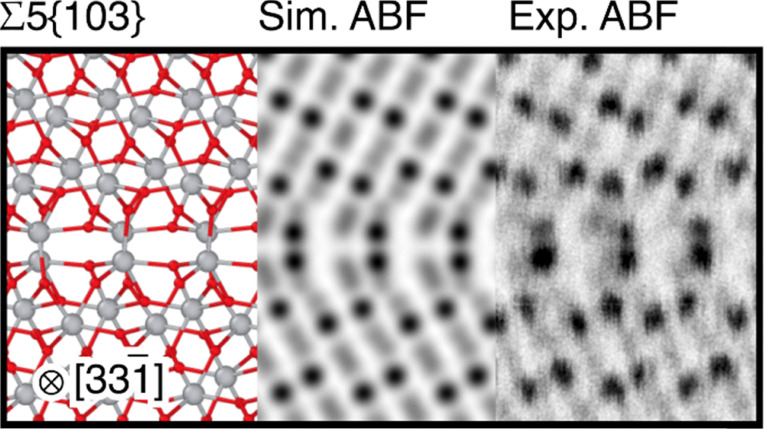
Structural model alongside simulated and experimental annular bright field (ABF) STEM images for the {103} symmetric tilt boundary in TiO_2_. Reprinted from ref. 75. Copyright 2021 American Chemical Society, licensed under CC BY 4.0.

Computational models provide invaluable insights into material design, but experimental validation is essential to build confidence in their predictive power. At a minimum, a model should predict a reasonable interface structure. Scanning transmission electron microscopy (STEM) enables atomic-resolution imaging of interfaces, with simulated microscopy comparisons often in excellent agreement^74^ (Fig. 5).

Care must be taken with STEM-based methods, however, as electron beams impart significant energy to a sample, which can have undesirable effects. Even materials that are, with care, stable under an electron beam may be unstable under the focused ion beam (FIB) that would ordinarily be used to cut thin samples.^76^ This reinforces the importance of computational methods in interpreting experimental observations. For example, in a study of titanium dioxide grain boundaries, EELS identified plentiful electron traps that were absent from computational models despite boundary structures being in good agreement. This disagreement prompted a more thorough analysis of the experiment, where it was found that the electron beam was creating large numbers of oxygen vacancies; inclusion of vacancies in the model introduced the observed electrontraps.^75^ Without computational support, incorrect assumptions would have been made about the nature of grain boundaries in the material.

The segregation of defects to a grain boundary is possible to observe in STEM through EELS, or even through contrast differences in the case of substitution defects with a large mass difference such as yttrium substituting aluminium sites.^77^ However vacancies are more nuanced, since there is no atom to directly measure and contrast differences will be negligible for low mass ions such as oxygen. The only experimental evidence for vacancies was indirect and therefore incorrectly attributed to the pristine boundary. Computational methods provided the detailed atomistic insight required for a revised, more truthful model.

Many materials, such as hybrid perovskites or lithium-battery materials, are also plagued by beam sensitivity. In some cases, it may not be possible to obtain good spatially resolved data experimentally. Fortunately, by rigorously confirming the accuracy of computational models where we do have good experimental references, we can improve our confidence in the situations where we don’t.

## 6. Challenges and future outlook

Explicit atomistic modelling is an important tool for probing solid–solid interfaces, providing atomic-resolution insight into properties that are difficult to obtain from experimental techniques alone. These calculated properties can guide the design of promising interface candidates before experimental synthesis and characterization. Interface energy and work of adhesion indicate whether two materials are likely to form a mechanically and thermodynamically stable contact. By varying the crystallographic plane, surface termination, and lattice strain, one can identify which interface geometries are most favorable for a given material pair. After mechanically unstable contacts are excluded, electronic structure becomes the next screening criterion. Band offsets and the DOS describe the electronic states available at the interface and the likelihood of electron or hole transfer. For example, in battery electrolyte–electrode interfaces, band offsets determine whether electron leakage or hole injection into the electrolyte is thermodynamically possible which can trigger decomposition reactions. PDOS provides more local information as localized states can indicate dangling bonds, undercoordinated atoms, or early signs of chemical reactivity. Bader charge analysis provides a complementary view of interfacial bonding and charge redistribution. For example, in catalytic heterostructures, charge transfer between the substrate and catalyst can change the electronic structure and selectivity of the active sites. In this way, interfacial charge transfer can be used as a descriptor when screening substrate materials or tuning surface reactivity. Interfacial stability calculations then test whether an interface is likely to survive under operating conditions. The electrochemical stability window defines the voltage range in which the interface is thermodynamically stable, while interfacial reaction energies quantify the driving force for mixing or decomposition at the contact. These quantities are especially important for solid-state battery interfaces, where a material pair can look favorable based on ionic conductivity and band alignment but still fail because the interface forms resistive decomposition products. Ion transport across the interface is often the descriptor most directly linked to device performance, although the relevant carrier depends on the application. Migration barriers from NEB calculations or diffusion coefficients from AIMD and MLIP-driven molecular dynamics capture the kinetic bottlenecks that often dominate interfacial resistance in ionic conductors. Together, these quantities define the main design requirements for promising interface candidates across energy storage, catalysis, and electronic materials such as stable adhesion, chemical stability, controlled interfacial charge transfer, and transport properties suitable to the target application.

Nonetheless, some fundamental challenges for explicit atomistic interface modeling still remain. First, constructing an interface requires matching lattices of two crystals, which usually introduces residual strain at the contact. The resulting properties also depend on the surface terminations and on the relative in-plane alignment of the two slabs. These choices create a large configurational space that is difficult to sample manually. Lattice-matching algorithms help reduce the number of possible models, but they do not remove the dependence on construction choices. In many studies, a single manually built interface is relaxed, rather than the interfacial structure being searched for systematically. Second, the gap between model interfaces and real interfaces remains a limitation. Real interfaces can be rough, non-stoichiometric, chemically intermixed, amorphous, or kinetically trapped in configurations determined by processing. MLIP-driven dynamics are making amorphous interphases and larger polycrystalline or grain-boundary models more accessible. However, these more complex models still require validation against experimental characterization, preferably under *operando* conditions, which is still rare. Another limitation is that the vast majority of current interface studies adopt a thermodynamic description and rarely use costly AIMD to classify the interface as stable or unstable. Yet most real interfaces are metastable, where kinetic passivation rather than thermodynamic equilibrium determines whether decomposition products continue to grow or form a self-limiting interphase. Capturing this behaviour requires dynamic simulations over nanoseconds and beyond. MLIPs are making this transition feasible by extending simulations toward nanoseconds and much larger cells while retaining accuracy close to the underlying first-principles data. Their reliability, however, depends on the chosen exchange–correlation functional and coverage of the training data. Because the configurational space at an interface is large, generating enough DFT reference data from explicit interface models alone can be expensive.

In future work, explicit interface modelling could be combined with MLIPs and targeted validation against experimentally measurable properties. Universal MLIPs can be fine-tuned using active learning on smaller bulk, surface, and amorphous structures that represent the local atomic environments expected at the contact. This could reduce the amount and cost of new training data needed for each interface chemistry and make it easier to test new material combinations without rebuilding a potential from scratch. Such potentials make longer and larger simulations accessible. They also make it more affordable to explore construction choices systematically, rather than committing to a single manually built model. This would allow more realistic interface models to be tested, together with more direct validation against experimental properties that require long simulations to converge, such as ionic conductivity and interfacial resistance. As more computed interface data accumulate, the extractable properties discussed in this Review could support database construction and larger-scale screening. In this way, explicit atomistic modelling can move beyond describing idealised interfaces and help predict which interfaces form, how they evolve under operating conditions, and which material combinations are most promising for energy storage, catalysis, and electronic devices.

## 7. Conclusions

In this concise Review, we have outlined the workflow of explicit atomistic modelling of solid–solid interfaces, from interface construction and atomistic simulations to the extraction of interfacial properties, the use of machine-learned interatomic potentials, and experimental validation. Explicit interface modelling enables the direct connection between interface structure, energetics, electronic properties, and ion transport, providing insights that are difficult to obtain experimentally alone. We have also examined the main challenges that this workflow still faces, from the choices made when the interface is first built to the cost of reaching realistic length and time scales. By combining atomistic simulations, machine learning, and experimental characterisation, explicit interface modelling can become a more predictive tool for understanding and designing solid–solid interfaces across energy storage, catalysis, and electronic materials.

## Conflicts of interest

There are no conflicts to declare.

## Data Availability

No primary research results, software, or code have been included, and no new data were generated or analyzed as part of this review.
